# Animal cultures matter for conservation, but also to animals

**DOI:** 10.3758/s13420-025-00700-4

**Published:** 2026-02-19

**Authors:** Simon Fitzpatrick, Kristin Andrews

**Affiliations:** 1 Department of Philosophy, John Carroll University, 1 John Carroll Boulevard, University Heights, OH 44118 USA; 2 Departments of Philosophy and Psychology, CUNY Graduate Center, New York, NY USA; 3 York Research Chair in Animal Minds, York University, Toronto, Ontario Canada

**Keywords:** Social learning, Animal culture, Conservation

## Abstract

A growing acceptance that many nonhuman animal communities have distinct cultures – group-variable patterns of behavior and information sustained over time by social learning – is beginning to reshape thinking about animal conservation. Culture, in this sense, can significantly influence how different populations interact with their environment and respond to environmental changes, and, therefore, has important implications for conservation. The literature on animal culture and conservation has led to valuable insights about how to protect endangered cultural animals. It has also led to some challenging questions. Should protecting animal cultural diversity become a new conservation goal, along the lines of preserving biodiversity? Should culture be an important consideration in prioritizing populations for conservation? Should we be designating animal “cultural heritage sites” for special protection, analogous to heritage sites of special significance for humans? This paper explores these questions and various arguments for preserving animal culture that have been offered in the literature. These include both instrumental arguments and arguments that suggest that animal cultures are of intrinsic value in their own right. These arguments raise important considerations, but they do not address all the ways in which animal cultures matter. We argue for more sustained attention to animals’ own interests in culture: animal cultures matter first and foremost because they matter to the animals themselves. Animals’ interests with respect to culture are not only about preserving practices or geographical locations, but include more abstract interests in protecting opportunities for agency, self-determination, and changing cultural traditions.

## Introduction

Human beings are not behaviorally homogenous. Not only do we vary on an individual level, we are also profoundly influenced by the different *cultures* in which we live – by the particular knowledge, information, and practices we acquire from, and pass down to, others around us. Culturally different communities might process food differently, select mates differently, and organize their groups differently. Different communities sharing the same space might have distinct cultural information about their shared environments, and react differently to the same challenges. Such group-level behavioral variation is taken for granted in the human case. Coming to see similar variation in nonhuman animal lifeways – recognizing that many animals *also* have their own local cultures – has revolutionized our understanding of the animal world, and it is beginning to influence thinking about wildlife conservation.

Like humans, many other animal species also have two inheritance systems: a biological inheritance system mediated by genes and a cultural inheritance system mediated by *social learning* (Avital & Jablonka, 2000; Richerson & Boyd, 2006; Whiten, 2005). Social learning is the ability to acquire behavior or information by observing or interacting with other agents or their products (Heyes, 1994). We now have evidence of social learning across vertebrate species, and there is increasing evidence for invertebrates, including insects (Whiten, 2021). Social learning enables the transmission of behavior and information from animal to animal horizontally (peer to peer) within generations, and vertically (parent to offspring) or obliquely (any older individual to any younger individual, or vice versa) across generations. Not all social learning produces culture. One can, for instance, socially learn a fleeting piece of information like the presence of a predator. Social learning is nonetheless necessary for culture. When individuals innovate a new behavior or learn new information individually, and the behavior/information is acquired by groupmates through social learning, this can lead to differences in the behavioral repertoires of different populations of the same species. Such differences can be stable over time, with each population transmitting its own behavioral “traditions” to the next generation. They may also evolve over time, with new behavioral traditions emerging via the contingencies of individual innovation and social transmission within populations. The culture of a particular population can, therefore, be understood as *all the information and behavior within the population that is sustained over time by social learning* (Richerson & Boyd, 2006; Schuppli & van Schaik, 2019).

Stable differences in behavioral repertoire between animal populations exist in a range of domains, including methods of foraging, tool manufacture and use, mating, migration, travel routes, and communication (Aplin, 2019; Boesch et al., 2020; van Schaik et al., 2003; Whiten et al., 1999; Whitehead & Rendell, 2015), but also in social dynamics (e.g., Kerjean et al., 2024; Sapolsky, 2006), social rituals and conventions (e.g., Perry et al., 2003; van Leeuwen et al., 2014), and possibly also social norms (Andrews et al., 2024; Westra et al., 2024). Methods for determining the extent to which these are sustained by social learning and thus cultural in nature include the method of exclusion, where researchers attempt to eliminate genetic and environmental explanations (e.g., asocial learning in response to different ecological conditions) for the relevant differences (Whiten et al., 1999; see also Schuppli & van Schaik, 2019), and statistical techniques, such as network-based diffusion analysis, which attempts to infer social transmission from the spread of behavior through social networks of individuals (Franz & Nunn, 2009; Hobaiter et al., 2014).

Some remain skeptical about animal culture in this sense (see Laland & Janik, 2006, for a review) – for instance, it can be difficult to completely exclude non-cultural explanations for behavioral diversity across populations, and, in many putative examples of culture in wild animals, social learning is inferred indirectly. Others insist on more demanding definitions of “culture,” requiring such things as cumulative change over time (e.g., Hill, 2009), or particular types of social learning such as active teaching or imitation (Galef, 1992) that remain controversial in animals (though see Whiten, 2022, on alleged cases of cumulative culture and imitation in nonhumans). There is, nonetheless, mounting evidence of diverse animal cultures in the sense at stake in this paper – stable population-variable behavioral repertoires sustained by social learning – across the animal kingdom (Schuppli & van Schaik, 2019; Whiten, 2021, 2022).

Although the prototypical examples of animal culture come from gregarious group-living animals, such as chimpanzees (Whiten et al., 1999) and whales and dolphins (Whitehead & Rendell, 2015), it is important to recognize that social learning can happen in many different kinds of contexts, including in comparatively solitary animals (Webster, 2023). Social transmission of behavior and information from parent to offspring is important in non-grouping as well as group-living animals; animals can learn important information from heterospecifics as well as conspecifics, and learning from others need not require direct social interaction. Animals can transmit information at a distance, for example, through chemical or auditory cues, or by leaving behind behavioral products, such as discarded tools or debris – from which others may learn new behavior. Unique socially transmitted behavioral traditions and even cultures may, therefore, exist in lots of surprising places, such as predator-prey interactions in particular geographic areas (Wooster et al., 2024), human training of guide dogs (Guillo & Claidière, 2020), and a variety of multi-species communities (Andrews et al., 2025; Sueur & Huffman, 2024). *Cultural animals –* species capable of social learning and for which there is some evidence of socially transmitted differences in behavioral repertoire across populations *–* therefore come in many forms.

The recognition that animals are not just products of genes, physical environment, and individual learning *–* that, in many species, culture matters, too *–* is beginning to reshape thinking within wildlife conservation science. Socially learned heterogeneity in behavior can significantly affect how animals interact with their environment, the roles they play in ecosystems, and how they respond to environmental changes, and, therefore, has important implications for conservation research, policy, and practice. Leading researchers on animal social learning and culture have been at the forefront of recent efforts to integrate understanding of these phenomena into wildlife conservation (e.g., Brakes et al., 2019, 2021, 2025; Kalan & Luncz, 2025; Kühl et al., 2019a; Laiolo & Jovani, 2007; Malherbe et al., 2025; Presotto et al., 2020; Ryan, 2006; Wessling et al., 2025; Whitehead, 2010; Whitehead et al., 2004, 2023).

This emerging literature on animal culture and conservation has led to valuable insights about how to protect threatened and endangered populations of animals that rely on social learning, but it has also led to some challenging new questions in conservation. For example, should protecting animal cultural diversity *itself* become a new conservation goal, similar to how conservationists have traditionally sought to protect genetic and other forms of biodiversity in animal populations? Should culture be an important consideration in delineating threatened and endangered populations and prioritizing them for conservation? And if so, how should we proceed? Moreover, to what extent should we see analogies between the protection of animal cultures and efforts to protect and preserve human cultural diversity and cultural heritage? For instance, should we be designating animal “cultural heritage sites” for special protection, analogous to heritage sites of special significance for human cultures?

In this paper, we explore these questions and the various arguments that have been offered in the literature for taking animal cultures and cultural diversity in animal populations to be worthy of conservation. These include both instrumental arguments for preserving animal culture and arguments that suggest that animal cultures are of intrinsic value in their own right. These arguments raise important considerations in favor of the conservation of animal cultures, but also give rise to some practical and conceptual concerns, which we will discuss. Moreover, they do not, in our view, fully capture the ways in which animal cultures matter from an ethical perspective. Here we argue for more sustained attention to the animals’ own interests; animal cultures matter first and foremost because they matter to the animals themselves. That idea has been hinted at in some recent discussions in the scientific literature – for instance, in the introduction to a special issue on animal culture and conservation (Brakes et al., 2025). The ethical implications of animal cultures are also receiving increasing attention from philosophers and other researchers in animal studies (e.g., Donaldson & Kymlicka, 2023, in preparation; Fitzpatrick & Andrews, 2022; Nussbaum, 2022; van Dooren et al., 2025). Drawing on some of these ideas, we defend the claim that, like humans, many other animals have ethically significant interests both as individuals and communities in practicing, participating in, and shaping the content of their cultural lives. Moreover, we argue that these interests with respect to culture are best understood in terms of thinking about culture as an *activity*, rather than in terms of particular practices or behaviors. The imperative to preserve animal cultures should, therefore, be primarily about protecting opportunities, agency, and self-determination for communities and the individuals that constitute them.

We begin by reviewing some of the ways in which animal culture has been seen as relevant to traditional conservation research and policy. We discuss proposals for conserving animal cultural diversity and the arguments that appear to motivate them. We discuss some of the practical and conceptual concerns that may be raised in response to these arguments, before presenting our own account of how and why animal cultures matter to cultural animals themselves.

### The relevance of animal culture to conservation research and practice

There are four straightforward proposals about how information about animal social learning and culture can help to support conservation efforts (Brakes et al., 2019, 2021). First, cultural behaviors can help us *see population structures and identify distinct populations of the same species for the purposes of conservation research* (Ryan 2006; Whitehead et al., 2004, 2023). Animal populations may overlap in geographic range, live in very similar environments, and exhibit little or no genetic divergence, yet exhibit differences in behavioral repertoire that warrant treating the populations as occupying distinct ecological roles. Vocal cultures in some whales and birds, for instance, play a similar role to that of linguistic dialects in differentiating human communities and have implications for other aspects of the behavioral repertoires of the populations (Hersh et al., 2022; MacDougall-Shackleton & MacDougall-Shackleton, 2001), such as sperm whales’ tendency to socialize only with other groups of the same vocal clan. Socially transmitted differences in foraging specialization (passed principally from mother to offspring) have also been identified as important in understanding population structure in dolphins and whales (Hersh et al., 2025; Whitehead et al., 2004). For example, three overlapping populations of killer whales in the northeastern Pacific exhibit different prey preferences: one specializes in salmon, another in marine mammals, and a third appear to specialize in sharks, despite all populations having potential access to these prey species (Ford & Ellis, 2014). These populations are genetically distinct, but there is no evidence that foraging specialization can be linked to genotype in killer whales. Rather, it has been argued that specialization likely began as different behavioral traditions that led to social segregation and reproductive isolation (Ford & Ellis, 2014; Riesch et al., 2012).

Second, an understanding of social learning and cultural differences is important for *assessing population resilience and adaptability*. Social learning can facilitate the evolution of new adaptive behavioral repertoires, such as when a keystone individual innovates a beneficial novel behavior and the behavior is socially transmitted through the population without individuals each having to re-innovate it. Because social transmission happens much faster than natural selection on genetic variation, it can help populations respond to quickly changing features of the environment. Cultural species may therefore be better equipped to survive anthropogenic environmental change (Whitehead et al., 2004). In some cases, new cultural practices have emerged in animal populations living in response to human activity that plausibly assist animals in surviving such encroachment. For instance, chimpanzees in Bossou, Guinea – an anthropogenically impacted population living alongside human villages and agriculture – developed new seemingly socially transmitted practices such as collective road-crossing and foraging for agricultural foods (Hockings et al., 2015).

Being potentially better equipped does not signify that the populations will flourish, however, as many of the behavioral changes arising in response to anthropogenic encroachments on great ape habitats also expose apes to new sources of stress, injury, and death (e.g., being hit by vehicles, exposure to pesticides, and attacks from farmers defending their crops), and may become maladaptive over time – for instance, leading to more hostile attitudes towards apes amongst the local human population (Gilbert & Kalan, 2025). As we know from the study of human culture, social learning can certainly produce maladaptive behavior (consider religious celibacy and suicide cults) and cultural conservatism, with populations sticking to existing behavioral traditions over generations, even when they are, or later become, maladaptive (Richerson & Boyd, 2006).

Cultural differences between populations of endangered species may therefore lead to differences in how they are impacted by anthropogenic effects, potentially requiring different conservation strategies (Brakes et al., 2019; Whitehead, 2010). For example, southern resident killer whales off the coast of British Columbia specialize in eating chinook salmon, whose numbers have been in decline, leading to increasing concerns about this critically endangered population as result of their apparent cultural conservatism (along with other anthropogenic threats), despite availability of alternative foods (Van Cise et al., 2024). Observations of southern residents killing but not eating marine mammals – a behavior that also seems to be socially transmitted – demonstrates the strength of this dietary conservatism (Giles et al., 2024). More recently, observations suggest the residents may be incorporating more diverse fish species into their diet, indicating the potential of new traditions that may help them survive the decline in chinook numbers (Van Cise et al., 2024). In cases like these, conservation research will be improved by considering the social dynamics that modulate which behaviors are socially transmitted, to understand the extent to which particular populations are adapting to changing environments.

A third context where social learning, culture, and conservation intersect will be in cases where animals need to *learn adaptive behaviors.* In breeding or rehabilitation and reintroduction projects, best practice guidelines increasingly recognize that the animals require the adaptive knowledge that wild populations have developed. For example, guidelines for the rehabilitation and release of great apes recommend that pre-release training should include experience with wild foods and methods of processing them, materials for building nests and tools, opportunities to travel on natural vegetation, exposure to natural predators and unknown wild conspecifics (Beck et al., 2007). Although some of this knowledge can be acquired via individual learning, social learning plays a key role for typical wild apes. Reintroduction projects, therefore, should consider the extent to which their success or failure may depend on leveraging social learning – for instance, providing naïve animals with appropriate cultural models from whom to learn – to facilitate the acquisition of adaptive behavioral repertoires (Greggor et al., 2025).

On the other hand, conservatism in social learning rears its head again when conservation programs introduce new elements into animals’ environments. Efforts to translocate animals to less anthropogenically disturbed habitats, or create wildlife corridors or other new travel routes through existing habitat, have sometimes been stymied by the animals themselves, when animals continued using traditional migration routes, even though the new ones appear safer to us. For instance, in a study of behavior at a Kenyan wildlife conservancy, more than 20 species persisted in using a crossing route into a neighboring habitat where there used to be a gap in a perimeter fence, even after the fence was removed and alternative routes made available (Dupuis-Desormeaux et al., 2018). Though there are many reasons why animals may stick to an established route rather than explore other options (e.g., scent cues may indicate its comparative safety), it is important to consider the possibility that some animals are sticking to a socially transmitted tradition. Remarkably, the number of species using the “ghost fence-gap” increased after being rendered obsolete, potentially indicating social learning across species. Such a potential role for socially learned traditions is important for gauging the likely effectiveness and potential side effects of conservation interventions, such as translocation.

Researchers have also argued that practices for sustainably managing populations of wild animals that are exploited by humans for food or sport should be informed by an understanding of from *whom* animals learn adaptive behavior. A common assumption has been that it is better for sustainability to target older animals for exploitation and protect younger animals for their reproductive potential. However, older animals often play an important role as keepers of community knowledge, such as seasonal migration routes, mating sites, and foraging strategies. The loss of older animals may thus have negative effects on population resilience via a loss of cultural knowledge (e.g., Slotte et al., 2025), leading to calls for special protection of cultural “keystone” individuals in both wildlife management and conservation more generally (Brakes et al., 2021; Kopf et al., 2024).

A final context in which conservation work may be informed by animal culture can be found in *mitigating human-wildlife conflict (HWC)*, such as crop raiding by chimpanzees and elephants, “man-eating” tigers, killer whales attacking yachts, or garbage raiding by bears or raccoons. Understanding the potential role of social learning in animal behaviors implicated in HWC is important for developing possible mitigation strategies. One way to eliminate undesirable behaviors perpetuated by social learning would be to break transmission chains by disrupting learning opportunities during critical periods. For example, black bears who raid garbage cans in Yosemite National Park have been found to learn the behavior from their mothers. To break the chain, parks close picnic areas when mothers with dependent young are in the area, eliminating either access to the garbage cans or removing the value of garbage cans during this period when the behavior is socially transmitted (Arbon et al., 2025). Understanding how human behavior is already impacting animal social learning and culture, and examining how human behavior may change or facilitate new (sometimes multi-species) traditions, can be a powerful tool for designing mitigation strategies.

These examples show how conservation and wildlife management research and practice can be informed by an appreciation of animal social learning and culture (Brakes et al., 2021, 2025), and that conservationists may need to recognize cultural animals as active participants in some wildlife conservation projects (Edelblutte et al., 2023).

### Conserving animal cultural diversity as a new conservation goal?

Another, less anodyne way in which animal culture has impacted discussion of conservation has been in calls to protect animal cultural diversity itself. Like the protection of human cultural diversity and heritage conservation, which focuses on human artifacts, languages, and traditions, the idea has been raised that certain cultural traditions and sites of animal culture should be targeted for conservation, even if the relevant species is not at risk.

For example, the bearded capuchin monkey is common across northern Brazil, and categorized as near-threatened (IUCN Red List), yet some populations of these capuchins are threatened due to legal and illegal expansion of agriculture, reducing the forest habitat of the relevant populations. These communities also display rare and sophisticated tool-use practices, including a variety of nut-cracking and crab-processing techniques, each different from the next community (Presotto et al., 2020). Reductions in community size, loss of resources and knowledgeable elders, and other disruptions to the conditions necessary for social transmission mean that unique cultural practices such as these may be lost, even if the relevant populations are not wiped out entirely, and without there necessarily being corresponding losses to genetic diversity or significantly enhanced risk of species extinction (Kühl et al., 2019a; Laiolo & Jovani, 2007). Calls have thus been made for preserving the cultural traditions themselves (Kalan & Luncz, 2025; Presotto et al., 2020).

On the face of them, such proposals raise the stakes for conservation policy beyond a traditional focus on species preservation. Whitehead et al. (2004, p. 434) note the likely concern, for instance, that “if we are at the stage of conserving nonhuman cultures, then the real conservation battles have already been won.” Of course, conservation biology is a “crisis discipline” (Soulé, 1985), heavily constrained by limited resources and limited influence over governmental and international policymaking. It is not practically possible to effectively protect every population of every species. In the face of that concern, there are different suggestions of how to integrate conservation of animal culture into the setting of conservation priorities with limited resources.

One approach draws on the existing practice of designating particular populations of animals below the taxonomic level of species as endangered and therefore priority targets for conservation action. A 1978 amendment to the US Endangered Species Act (1973; United States Congress, 1978) (and similar legislation in Canada and other countries) enabled the designation of distinct population segments as “species” for the purpose of applying the legislation (Hoelzel, 2023; Ryan, 2006). The concept of *evolutionarily significant units* (ESUs) arose (in part) to facilitate this designation process. Though a major goal is to identify and protect distinct populations of a species that are most at risk, the idea behind ESUs is also to identify particular populations that have some special significance for maintaining biodiversity and “represent an important component in maintaining the evolutionary legacy of the species” (Waples, 1991, p. v) – populations with unique evolved features that would be lost to the world with the extinction of the population, giving rise to a significant loss to the ecological diversity of the species. Potential for *future* evolutionary change is also part of this notion of “evolutionary legacy.” Though definitions vary (Hoelzel, 2023), ESUs have historically been grounded in criteria of genetic differentiation, geographic isolation (hence, little interbreeding with other populations), and locally adaptive traits (suggesting independent evolutionary trajectory). The classic example is Pacific salmon (Waples, 1991). Several distinct populations of chinook salmon in the USA and Canada have been designated as protected populations, yet others are exploited by commercial and recreational fishing.

In light of this, it has been proposed that certain culturally identifiable animal populations be designated as “culturally significant units” (CSUs) to potentially be prioritized for conservation action (Ryan, 2006). The idea is that unique socially transmitted behavioral repertoires that differentiate a population from others can be used, in some cases, to identify conservation units that are at particular risk. Moreover, insofar as such repertoires have their own cultural evolutionary history, they may have similar claim to “the evolutionary legacy of the species” to the criteria for ESUs. Hence, CSUs should also be considered in setting conservation priorities. Kühl et al. (2019a) and Presotto et al. (2020) appeal to the CSU concept to argue that the protection of cultural diversity should be incorporated into conservation planning for wild chimpanzees and bearded capuchins, respectively – for instance, by conserving their natural resources and habitats where tool-use traditions arise (see also Kalan & Luncz, 2025).

Another option is to broaden the ESU concept to potentially include culture alongside traditional criteria (Whitehead et al., 2023). In this way, cultural repertoire can be seen, in at least some instances, as contributing to the discreteness of potential conservation units and/or to evolutionary significance. On evolutionary significance, Whitehead et al. write:

Behavioral differences should be considered evolutionarily significant if they are important demographically, ecologically, or perhaps in other ways (such as dealing with environmental change), and if one community was lost, the behavioral information that it possessed could not easily be reconstituted. (Whitehead et al., 2023, p. 3).

Whitehead et al. review how and where this standard might be met. It is important that the relevant behavior be stable across multiple generations, mark out behaviorally cohesive, discrete population units, either qualitatively (for instance, presence vs. absence of the relevant behavior in one unit compared with another) or quantitatively (for instance, common vs. rare), and indicate a distinct ecological role that could not easily be reconstituted by another population of the species (i.e., the population unit does something unique in the ecosystem, such as migrate to forage in a particular geographic location, that is unlikely to be independently replicated by a different population of the same species). They suggest, for example, that socially transmitted song or call structure in whales can help delineate discrete populations and may be evolutionarily significant when influencing how individuals interact, such as marking out “us versus them,” and hence influencing mating behavior and the flow of heritable information. Similarly, stable socially learned migrating behavior and dietary preferences in whale, fish, and bird populations may count as evolutionarily significant, insofar as they are not purely environmentally driven and the loss of the population with that cultural knowledge would lead to irreversible loss of the relevant species from particular habitats and roles in ecosystems.

Finally, a third suggestion calls for the establishment of animal “cultural heritage sites:”

We suggest that, for chimpanzees, specific interventions are needed to protect their natural resources and tool-use sites in order to maintain behavioral plasticity and safeguard their capacity for cultural evolution. Therefore, we anticipate the necessity for a new concept, “chimpanzee cultural heritage sites,” with which the behavioral and cultural diversity of this species might be recognized and protected. Such a concept could easily be extended to other species exhibiting a high degree of cultural variability, such as orang-utans and whales. (Kühl et al., 2019a, p. 1455)

The invocation of “cultural heritage” naturally suggests analogies with the protection of human cultural heritage and that historical significance matters alongside evolutionary significance. It also points to a potential elaboration of the existing framework of UNESCO for listing World Heritage sites, which include “extraordinary natural places on the planet, characterized by their natural beauty or outstanding biodiversity, ecosystem and geological values,”^1^ to include some sites of animal culture – as recently argued by Hynninen et al. (2025). West African chimpanzee nut-cracking sites, for instance, have an important place in the history of the study of animal behavior and tool use, and the use of stone tools by chimpanzees in this region can be dated back several thousand years (Mercader et al., 2007).

In addition to the considerations discussed in the previous section, these proposals about incorporating the preservation of animal cultures into conservation prioritization have had some policy impact (see Brakes et al., 2019, 2021; Wessling et al., 2025), notably via the UN Convention on the Conservation of Migratory Species of Wild Animals (CMS). A 2014 resolution adopted by parties to the convention led to the establishment of a scientific working group on the conservation implications of animal culture and social complexity. This led to a 2017 “Concerted Action for Sperm Whales (*Physeter macrocephalus*) of the Eastern Tropical Pacific,” which focused on four clans of sperm whales identified by their vocal repertoires rather than by their genome (CMS, 2017). This was followed by a 2020 “Concerted Action for The Nut-Cracking Populations of the Chimpanzees (*Pan troglodytes versus*) of West Africa,” again identifying populations of the critically endangered Western chimpanzee subspecies as priority targets for conservation action by virtue of their nut-cracking behavior, which is not present across the whole range of the subspecies (CMS, 2020). That was replaced in 2024 by a broader “Concerted Action for Chimpanzee (*Pan troglodytes*) Behavioral Diversity and Cultures,” targeting all four subspecies of chimpanzees (CMS, 2024). There has also been increasing collaboration between CMS and the International Union for the Conservation of Nature (IUCN) in this area (see Wessling et al., 2025).

The claim behind these proposals is that animal culture offers more than just new tools for traditional conservation work; animal culture and cultural diversity should be a new conservation goal itself. In the next section, we articulate what we take to be the underlying reasoning motivating this idea. As readers will probably already be able to tell, a variety of different considerations seem to be at play, reflecting the diverse kinds of values that conservationists appeal to in their research and advocacy. In the section *Pitfalls*, we highlight some practical and conceptual concerns that may be raised in response to these arguments.

## Instrumental and intrinsic reasons for preserving animal cultural diversity

When looking at these different proposals for conserving animal cultures and cultural diversity, we see that both *instrumental* and *intrinsic* reasons have been given for why animal culture should be protected and thus considered in conservation policy.

### Instrumental reasons

Instrumental reasons for preserving animal culture do not take such preservation to be an ultimate conservation goal, only a secondary goal: a *means* for achieving existing conservation goals. Such instrumental reasons seem to fall into three categories:

1. Preserving animal culture to support species survival;
2. Preserving animal culture to support human interests in healthy ecosystems; and
3. Leveraging animal culture for its public relations value in drawing resources to conservation work. We briefly describe the logic of each.

#### Preserving animal culture to support species survival

Cultural diversity in animal populations is important for population resilience and adaptability, insofar as it means that populations have, or have a greater chance of developing through cultural evolution, an adaptive toolkit for surviving in changing environments. For familiar Darwinian reasons that evolution by natural selection requires *variation* and that such variation may support species survivability in the face of environmental changes via adaptation, preserving cultural diversity should be seen as a means for facilitating the survival of species in the same way as preserving genetic diversity. An important implication of this argument is that conservation should not just focus on overall numbers of animals, but also take into account demographics, community cohesion, available resources (e.g., nut trees for nut-cracking chimpanzee populations), and other variables relevant to maintaining social transmission of adaptive information and potential for cultural evolution (Kühl et al., 2019a).

#### Preserving animal culture supports human interests in healthy ecosystems

Standard anthropocentric arguments for biological conservation emphasize its importance for human interests – for example, the concept of “ecosystem services” (Daily, 1997) – given the connected nature of ecosystems and the impact of ecosystem disruption on human health and economies. Another instrumental argument for conserving animal cultures trades on this idea. For instance, Sueur writes, “the destruction of animal cultures can disrupt ecosystem services that are vital for human health. For instance, if a keystone species loses its cultural knowledge, it could lead to a decline in its population, which in turn could destabilise the ecosystem and reduce the services it provides, such as water purification, pollination, and disease regulation” (Sueur, 2022, p. 98). Again, the idea is that conservationists need to look beyond conserving genetic diversity and population numbers to also include cultural preservation, not just for purposes of preserving the relevant species but also for anthropocentric reasons. Sueur therefore argues that animal cultural preservation should be incorporated into the World Health Organization (WHO) One Health concept,^2^ an interdisciplinary approach that emphasizes the role of connected ecosystems, and that the health of environments and nonhuman species is vital to human health and wellbeing.^3^

#### Leveraging animal culture for its public relations value

Another instrumental argument depends on the public relations value of animal culture for generating resources for conservation work, similar to the long-running strategy of designating particular species, such as the giant panda or Bengal tiger, as “flagship species” for special protected status. Just as public interest and affection for such flagship species can be leveraged to bring in greater financial resources and public support for conservation more generally, so may public interest in stories about animal culture. For example, Laiolo and Jovani (2007, p. 5) write, “Conservationists could [...] use the appeal of animal culture as a tool that is relatively easy for the general public to understand, compared with, for example, genetic variability, to direct conservation planning, in a similar way that emblematic species, such as birds of prey, are used to publicize the conservation of diverse ecosystems.” Similarly, Carvalho et al. (2022, p. 4) note that “There is potential value in using nonhuman culture to attract public interest and elicit empathy toward species of concern,” and Sueur (2022, p. 99) writes, “By highlighting the cultural aspects of species, conservationists can create stronger emotional connections between the public and wildlife, which can lead to increased funding and political support for conservation initiatives.”

### Intrinsic reasons

In addition to these instrumental arguments, two distinct arguments have been provided for animal cultures and cultural diversity having intrinsic value in their own right. The first articulates an argument for why the biodiversity concept should be expanded to include socially learned behavioral diversity. The second draws on analogies with human culture that is worthy of heritage protection.

#### Value of behavioral diversity

The conservation of biodiversity is often articulated as the central goal of conservation. Protecting the persistence and adaptive potential of species is one way to protect biodiversity, and, as described above, protecting animal cultural diversity has been argued to be a means to that end. However, insofar as biodiversity is understood as diversity among living organisms at all levels of organization, including diversity of ecosystems and species, but also genetic and phenotypic variability, then animal behavioral diversity *itself* is also an important part of biodiversity, even when it is not straightforwardly correlated with (or a means to preserving) genetic diversity or diversity of species. According to this logic, animal cultures should therefore warrant conservation in their own right as part of efforts to conserve biodiversity. Importantly, the tradition of conservation biology following Soulé (1985) holds that biodiversity has intrinsic value, the conservation of which should constitute an end in itself, independent of the benefits to humans (e.g., ecosystem services) derived from biodiversity (reflected, for instance, in the UN Convention on Biological Diversity, 1992). In this sense, then, cultural diversity has intrinsic value as a manifestation of biodiversity (e.g., Wessling et al., 2025; Whitehead et al., 2004, 2023).

#### Analogy with human cultural preservation

Human culture is as much part of biodiversity as animal behavioral diversity, but imperatives to preserve human cultural diversity and heritage predate conservation biology and the evolutionary study of human and animal behavior, and typically derive from very different considerations. National and international institutions such as UNESCO exist to protect and promote human cultural diversity and heritage not for reasons of biodiversity, but because human cultural diversity is seen as a good in its own right.^4^ As we have seen, analogies between human and nonhuman culture therefore invite similar arguments for preserving animal culture. McGrew (2003, p. 438), for instance, writes, “if nonhumans have culture in any form, then we must be concerned with cultural survival,” and Whitehead (2010, p. 334) writes “as has been repeatedly argued for human cultural elements such as languages and architecture, cultural diversity should be conserved as a principle.” We take this to also be an animating idea behind animal “cultural heritage sites” (Kühl et al., 2019a).

### Pitfalls

We agree with many of the ideas behind the arguments just described for caring about conserving animal cultural diversity. However, they raise both practical and conceptual worries about how to implement conservation policy with them in mind. Some of these have already been acknowledged in the literature (e.g., Carvalho et al., 2022; Wessling et al., 2025), but we think that there are some other concerns that need to be brought to light. Moreover, these arguments overlook what we see as the most important reason why animal cultures should matter from an ethical perspective.

#### Human biases

First, one worry about using the cultural repertoire of a population as a potential reason to prioritize it for conservation over others – an idea common between several of the instrumental and intrinsic arguments – is that this may lead to an emphasis on preferentially protecting populations with behaviors that are more eye catching to humans. For example, in the case of conserving endangered wild chimpanzee populations, Hockings and McLennan (2019) raise the concern in response to Kühl et al. (2019a) that populations trying to survive in more human-altered landscapes and those who have direct behavioral interactions with humans will naturally get less attention than more “pristine” populations who more closely live up to the stereotype of wild chimpanzees and thus have more idealized “wild cultures.” Populations in more anthropogenically disturbed habitats, however, face greater threats. These populations have also developed novel cultural practices, often in response to human activity, and sometimes *with* humans as cultural participants, but these are likely to appear to be less “worthy” of protection (though see Gilbert & Kalan, 2025, on the potential maladaptive consequences of some of these new behaviors) when applying notions of cultural significance. Kühl et al. (2019b) reply that their claim is not that such populations are less worthy, but rather that behavioral diversity and historical significance are considerations that should be integrated with traditional considerations for setting conservation priorities, such as population size, genetic diversity, and population viability.

Strictly speaking, such biases run against the logic of several of the instrumental and intrinsic arguments. For example, it is not clear why more recent or more anthropogenically influenced cultural practices should be seen as having less intrinsic value in terms of manifesting biodiversity or aesthetic value, say. However, a concern is that, in practice, thinking about what counts as contributing to such things as biodiversity or evolutionary or cultural “significance” is often centered around a “non-anthropogenic” ideal – for instance, prioritizing “native” species over those introduced into ecosystems intentionally or unintentionally by human activity, even when ecosystems have adapted to the presence of non-native species (Castelló & Santiago-Ávila, 2023). Of course, ultimately, humans are only going to take action to conserve what they care about (if that), but it is nonetheless important to be mindful of potential biases and the risk of introducing ethically unjustified differentiations between populations when it comes to using cultural repertoire in setting conservation priorities. This worry was one reason why the CMS concerted action for nut-cracking chimpanzees (CMS, 2020) was replaced by a broader concerted action going beyond the West African chimpanzees and looking at chimpanzee behavioral diversity more generally (CMS, 2024; Wessling et al., 2025).

#### How to prioritize cultural behaviors to protect

There are also evidential problems with applying concepts like evolutionary significance to pick out which populations to prioritize in virtue of cultural repertoire. ESUs rely on the condition that an evolutionarily significant unit has distinctive genetic, phenotypic, or ecological features that could not easily be reconstituted with the loss of the population (Whitehead et al., 2023) – hence, threats to such populations constitute unique threats to biodiversity. In the case of cultural behavior, it is difficult to establish the counterfactual: could the behavior be reconstituted in a separate or new population were the original population to be lost? We might have strong suspicions that this will not be the case, but it is much harder to be as confident about this with socially learned behavior compared with the reconstitution of genetic features. How much normative weight should be placed on this in any case? Many of the greatest feats of human culture have been independently invented multiple times – the wheel, agriculture, pyramid architecture, and so forth – and it is currently controversial, for example, whether or not many features of chimpanzee tool use cultures (including stone tool nut-cracking) could reasonably be expected to be independently re-innovated by culturally naïve chimpanzees (Tennie et al., 2020; Whiten, 2022). Should the possibility of reinvention weaken the case for acting to protect these populations? By the logic of prioritizing populations in virtue of unique and difficult to replace contributions to biodiversity it would seem so. The theoretical possibility of reinvention may be counterbalanced by the fact that time is not on the side of endangered long-lifespan species, but this point does raise the question of whether such features of the relevant cultural practices *should* guide prioritization, even if only one factor amongst many.

The instrumental contribution of particular cultural practices to future adaptive potential and species survivability is also often difficult to establish. It has been argued that nut-cracking in chimpanzees helps the relevant populations utilize forest habitat less abundant with more typical chimpanzee foods (Yamakoshi, 1998), but the empirical case for that claim is not clear (Carvalho et al., 2022; Koops et al., 2014). The potential link with impacts on human health or other ecosystem services from the loss of the behavior is even more difficult to determine. Even when instrumental contribution to adaptive potential or relevant ecosystem services can be established, the fact that cultural behaviors can also become maladaptive with changes to the environment also arguably raises the stakes for when particular cultural behaviors can be used as a basis to selectively act to protect particular populations when concepts such as “evolutionary legacy” are invoked. As Carvalho et al. (2022) argue, making conservation policy subject to such empirical uncertainties – also including the difficult problem of determining whether a particular behavior is socially learned in the first place – is unfortunate in the face of the severity of the threats to many of the relevant populations.

In addition, as van Dooren et al. (2025) note, no population of animals develops its culture in isolation from the other organisms and features of the environment with which it interacts. Culture is pervasively multispecies (Andrews et al., 2025) and the track record of research in this area also suggests that there is so much animal culture out that we do not yet know about, all of which contributes to biodiversity. How can we pick out, in a principled way, which cultural behaviors are most “significant” and “worthy” of focused conservation effort?

#### Risks of a new cultural great chain

With respect to the invocation of concepts like “cultural heritage,” there are also dangers lurking here harkening back to racist nineteenth century views of what qualifies as “cultured,” in contrast to “savage” human societies. At the height of the British empire, “culture” referred to the idea that some few human societies had reached a “higher” status, replete with institutions, arts, and laws and norms that reflected the values of world leaders of the time – justifying further colonial expansion. It took anthropologists such as Franz Boas to undermine this ranking of cultures on a normative scale from “lower” to “higher.” Boas writes, “the grand system of the evolution of culture, that is valid for all humanity, is losing much of its plausibility. In place of a single line of evolution there appears a multiplicity of converging and diverging lines which it is difficult to bring under one system” (1904, p. 522). Boas’ rejection of a normative hierarchy of cultures parallels the Darwinian rejection of the Great Chain of Being and a normative ranking of “higher” and “lower” species, though this kind of thinking does, unfortunately, live on in snobbish attitudes about which and what aspects of human cultures deserve to be preserved for posterity. We emphasize that no one in the animal culture conservation literature is arguing for such a ranking of “higher” and “lower” cultures, but the concern is that talk about cultural heritage and significance can easily slide, unintentionally, into an illegitimate normative ranking of cultures, and, again, risks introducing biases favoring particular types of cultural practice that happen to be eye catching to humans.

More generally, one thing that seems curiously missing from many of the arguments described above are the animals themselves. In trying to advocate for the relevance of their research to conservation and for the animals that they study, animal culture researchers obviously have had to adapt their language and arguments to their audience, the linguistic norms of scientific journals, and the constraints of existing conservation policy frameworks, such as the US Endangered Species Act. It is unsurprising, therefore, that the focus of the work we have cited is on the relevance of animal culture to questions of biodiversity, species preservation, ecosystem services, and strategic public outreach, given those are the lingua franca of conservation biology and conservation policy circles. Though the literature on animal culture and conservation is intended to challenge many of the underlying assumptions of mainstream conservation biology and policy thinking (e.g., calling for conservationists to look beyond genetic diversity, recognize that populations of the same species may not be behaviorally homogenous, and take account of the role of social transmission of behavior when considering interventions such as translocation; Brakes et al., 2021, 2025), this approach still inherits the ethical problems of mainstream conservation biology that have been pointed out by critics (see, e.g., Castelló & Santiago-Ávila, 2023; Edelblutte et al., 2023; Rizzolo & Bradshaw, 2021) – most importantly, the tendency to overlook the interests and wellbeing of the animals involved (both as individuals and communities). Even those who have stepped beyond the traditional language of conservation biology and have sought to make the analogy with the preservation of human culture articulate cultural conservation with respect to animals largely in the abstract. Animal cultures are depicted as something to be preserved almost independently of the animals manifesting them, without attention to why the conservation of human culture is important for the wellbeing of human beings. A complete understanding of why animal cultures matter and why humans may have obligations to take steps to preserve them (and what that should mean in practice) has to include consideration of the animals themselves.

That said, that animal cultures might also matter *to* cultural animals has been floated in some discussions in the scientific literature. For instance, in the introduction to a recent special issue on animal culture and conservation, Brakes et al. (2025, p. 10–11) write: “we caution against simply assessing the merit of cultural diversity in other species for its mechanistic value or utility to conservation alone… animal cultures may have intrinsic value, not just to those human cultures that observe and interact with them, but perhaps for the animals themselves” (see also Whitehead et al., 2004, p. 434, for similar remarks). Philosophers and researchers in animal studies have also recently presented arguments in this direction (e.g., Fitzpatrick & Andrews, 2022; Donaldson & Kymlicka, 2023, in preparation; Nussbaum, 2022; van Dooren et al., 2025). In what follows, we develop a case for how and why harms to culture should be seen as harms to animals themselves, building on the literature on the concept of cultural rights for human beings, and draw out the implications of this perspective for the conservation of animal cultures.

### Harms to culture are harms to animals themselves

Why should humans care about human cultural diversity and the preservation of human cultural heritage? These ideas are often taken for granted, but they have been the source of much controversy in ethics and political philosophy, and in human rights law, especially in the context of debates around multiculturalism, liberalism, and the relationship between Indigenous and minority communities and national governments (e.g., Kymlicka, 1996). For example, to what extent should claims of cultural tradition or heritage be respected when it conflicts with individual liberty: for example, the rights of girls and women in historically patriarchal cultural communities, or the rights of LGBTQ+ individuals in religiously conservative communities? To what extent can Indigenous peoples claim autonomous control over their ancestral lands from national governments who may wish to exploit those lands for economic or other purposes they may argue to be in the broader public interest? Similarly, what is the goal of cultural heritage protection, such as the preservation of sites and artifacts of historic cultural significance? And what role should present generations of relevant communities historically associated with these sites/artifacts have in that endeavour (Poulinis, 2010)? We cannot go into all the ins and outs of these debates, but we do describe an approach to thinking about some of these questions that we think is helpful for understanding how and when harms to human culture are harms to humans.

International human rights documents, such as the International Covenant on Economic, Social and Cultural Rights (1966), recognize so-called “cultural rights” for all human beings (Donders, 2019). Cultural rights are here understood to be *additional* to traditionally recognized rights in the liberal political tradition, such as rights to freedom of speech and conscience, free movement and association, political representation, and so forth. For example, the International Covenant on Economic, Social and Cultural Rights articulates the “Right of everyone to take part in cultural life” (Article 15, paragraph 1(a)). This right is elaborated by the UN Committee on Economic, Social, and Cultural Rights (General comment No. 21, 2009), which emphasizes participation, access, and contribution: everyone has the right to participate in the cultural life of their community in whatever way they may choose; everyone has the right to access information and knowledge related to their particular cultural heritage; and everyone has the right to contribute to the cultural life of their community. These rights impose obligations on governments to refrain from interfering with the right of participation, prevent third parties from engaging in such inference, and also provide financial and other resources towards cultural education, creating infrastructure for the promotion of cultural expression and diversity, and the protection of cultural artifacts, heritage sites, and so forth.

But why think that humans have rights to culture and what do such rights protect? Cultural rights have been controversial (Donders, 2019). For instance, critics worry about cultural excuses for human rights violations or undue deference to problematic cultural traditions, such as forced marriage or forced prostitution. Holder (2008), however, argues that a plausible view of what they mean has emerged over recent decades: the “activity” conception. Culture in this conception is understood as something that people *do –* an activity *–* that is fundamentally linked to their dignity. One cannot recognize and respect the dignity of individual human beings without taking into account that humans have ethically significant interests as members of cultural communities who have created a particular shared way of living. Hence, cultural communities can be treated as rights holders insofar as the individuals who make up those communities share relevant interests as a collective and/or their individual wellbeing is closely intertwined with living a particular collective way of life. However, given that culture is here understood as an activity, cultural rights therefore attach to individuals and communities of individuals with shared interests in pursuing that activity, not to particular cultural practices themselves. They have the typical restriction that liberal rights theories place on individual rights: that the exercise of one’s rights should not interfere with the rights of others.

Cultural rights are, therefore, not undermined by the fact that many features of human cultures are indeed profoundly harmful (nor do they entail cultural relativism), given that they do not protect practices themselves. Rather, they are about opportunities, agency, self-determination, and dignity for communities and the individuals that constitute them *–* their ability to determine the content of their social lives *for themselves* and all that entails. We obviously *also* care about the historical and aesthetic value of cultural practices and artifacts (e.g., languages, rituals, artworks, etc.) and seek to also preserve those things for such reasons, but the key idea is that a primary reason for caring about culture and cultural diversity is because we care about the freedom and dignity of living *people*. Attacks on culture, such as restricting the ability of people to continue to speak their ancestral language, or the destruction of a religious shrine, are problematic not just because of losses to historical or aesthetic value, but more importantly because they are attacks on people.

That what is protected by cultural rights is something that people do and should have some freedom over makes clearer the distinctive implications for Indigenous communities (Holder, 2008). Cultural rights are invoked to justify claims to autonomous control over ancestral lands and resources, potentially overriding claims of national economic interest in exploiting those resources. The activity conception makes sense of this insofar as one cannot respect the ability of a community to determine the cultural content of their lives without them having a say over the material resources central to their distinctive way of life. It also avoids the charge of cultural essentialism, and the problem of defining “the culture” of a people that is not undermined by changes to their way of life over time, or implies that the goal is somehow to *freeze* a culture in place *–* treating real living people and communities as museum pieces to be preserved for the curiosity of others.

A similar view has emerged in the context of conservation of “living cultural heritage sites” (Poulinis, 2010) *–* sites of historical cultural significance that maintain their original function and where the community of people historically associated with the site retain their use and proximity to it. At an institutional level, heritage site conservation around the world has tended to focus on preserving the material fabric of the site, with particular attention to the positive values associated with the site by the people who created it. However, as Poulinis (2010) argues, in practice, this has tended to disconnect the project of heritage conservation from the people historically connected with it, treating the site as a thing of the past, the “authenticity” of which needs to be maintained, discontinuous from the people of the present. A living heritage approach, instead, focuses on “maintain[ing] the continuity of the (present) community’s association with a site. The protection of the fabric is placed within the maintenance of the continuity of a community’s association with a site” (Poulinis, 2010, p. 180). A key part of this approach is that the “core community” becomes the primary stakeholder, whose interests trump other stakeholders. Again, this reflects the idea that preserving culture and heritage should really be seen as primarily about protecting the freedom and dignity of people of the present.

We argue that a similar perspective should be adopted towards animal cultures. The arguments described previously for protecting animal behavioral diversity, such as the intrinsic value of behavioral diversity as biodiversity or its instrumental contribution to species survivability, overlook something extremely important, namely, that harms to culture are direct harms to cultural animals themselves. Though, to date, there has been little scientific work looking directly at a potential relationship between social learning, culture, and animal welfare, we have argued elsewhere that cultural animals likely have distinctively *cultural needs* and that the exercise of capacities for culture should be seen as an important component of their wellbeing (Fitzpatrick & Andrews, 2022). Research on social deprivation (e.g., the notorious experiments of Harlow and colleagues; Harlow et al., 1965) has shown that social animals have fundamentally social needs, and isolation typically produces very poor welfare outcomes. The literature on animal culture suggests that an individual cultural animal *–* or even a group of animals *–* isolated from their cultural traditions would be at a loss, even when living in an appropriate habitat. They not only lack information about basic survival skills, such as what to eat or where to sleep, but they also lack a network of social practices that scaffolds social interactions, such as learned communicative signals, norms that structure relationships, rules about social hierarchies, and so forth. For animals that have evolved to depend on social learning, it is also not unreasonable to entertain that they are fundamentally motivated to learn from others and potentially be models for others to learn from *–* hence, they are harmed when deprived of opportunities to exercise and develop these capabilities (Nussbaum 2022). As van Dooren et al. (2025, p. 13) aptly put it, “conservation of the organism without culture *–* just like that of the organism without environment *–* can hardly be called conservation at all.” Without culture, a cultural animal is only half of who they *should* be.

Some may worry that attributing something like cultural rights to animals is an anthropocentric projection: surely, animals do not have the kinds of interests in culture that are reflected in human museums and monuments to culture and cultural expression; as far as we know, animals do not make a conscious effort to preserve or disseminate their own local cultures. Of course, culture may not matter to animals in the conscious and reflective way that a religious temple or ritual may matter to a human being, but explicitly expressed and reflected upon beliefs and values are just one part of human culture that matters to human beings, and culture does not just matter to us for intellectual reasons. Much of human culture is implicit and rarely articulated, at best only dimly understood by the relevant communities (Henrich, 2015), and never makes it into a museum or documentary *–* think of social norms governing things like queuing or how close one should stand when talking to someone else, mannerisms, accents, and so forth. These are all just as much part of a shared way of life that reflects the collective self-determination of a community of human beings and become interwoven with the determinants of their wellbeing as are expressed values and belief systems and the sorts of artifacts that end up in museums. The activity conception we have described emphasizes that culture matters because it reflects the shared way of living that a self-determining community has created for itself *–* reflectively understood or not *–* and we should care about the ability of a community to maintain their (cultural) self-determination. There is no good reason for thinking that only matters because (or when) it is something of which people are consciously aware. Animals do not have to build museums to their cultures for culture to still be part of the determinants of their wellbeing.

Another potential concern about applying something like cultural rights to animals is that animal cultures may often be hierarchical, with only a few members of the community able to exert significant influence over its norms and social culture. Consider, for instance, the social hierarchies of primate groups (which appear to have socially learned elements; see, e.g., Sapolsky, 2006) that benefit some group members, but harm others, such as inducing stress and limiting the opportunities and agency of subordinates. As we have seen, assertions of human cultural rights are generally only seen as legitimate under international law when all members of the relevant community are able to participate in and shape the community’s culture (Donders, 2019). However, similar inequalities in access and influence do not undermine the claims to cultural rights of individuals in the human case, and it is the recognition of the fact that human cultural practices and social structures may often perpetuate harm and undermine the freedom of dignity of community members that has shifted the emphasis in thinking about what cultural rights protect away from practices themselves to the activity conception (Holder, 2008). Individuals and communities of cultural animals should still be seen as having rights to cultural autonomy and self-determination *–* rights to *do* and *shape* culture *–* even if they happen to have cultures that may perpetuate harm and inequity. Cultural change in animal populations may therefore often be desirable, but, as in the human case, it will ultimately be up to the animals themselves to determine the contents of their cultural lives. This is not to say that there should never be outside influence, though, or that animals should always be left alone to do what they will. Cultural self-determination can cause conflict between cultural groups *–* human and nonhuman *–* and the self-determination rights of individuals and groups end where those of others begin. Again, as in the human case, the question then becomes what processes of negotiation are possible with other communities who have different values or interests?

What we suggest, then, is that instead of talking about conserving animal cultures or behavioral diversity, we should focus on conserving the *autonomy* of animal populations. This better reflects what we see as the most important reason why we should consider the loss of animal cultures concerning. Losses of unique practices like tool use practices in bearded capuchins (Presotto et al., 2020) or gestural dialects in chimpanzees (Malherbe et al., 2025) are things that we should care about primarily because they are symptoms of losses to the autonomy of these communities of animals: they are becoming less able to pursue their distinctive shared way of life and that is *a harm to these animals*. Suppose, for instance, that these practices disappeared, not because of anthropogenic disturbance, but because of non-anthropogenic processes of cultural evolution. That should surely be much less concerning, even if it meant a loss to biodiversity or some loss to cultural “heritage” (say the traditions, like most animal cultural behaviors, had never been documented by humans). It is the fact that the animals did not decide for themselves (albeit in an implicit and socially distributed way *–* note that even in humans cultural changes are rarely intentionally guided (Henrich, 2015)) to leave the tradition behind that is most significant. Similarly, one should not have to demonstrate that the behaviors could not be reinvented independently by another population, that they serve some key adaptive function, or that there are likely to be some downstream consequences of their disappearance for human interests.

Our argument chimes with broader critiques of mainstream conservation biology, which hold that it tends to arbitrarily subordinate diverse sources of value in nature to often heavily idealized values like “biotic diversity,” “evolutionary legacy,” “ecological complexity,” “ecosystem integrity,” and so forth (e.g., the tradition of Soulé; see Castelló & Santiago-Ávila, 2023). Interventions such as targeted hunting or culling, translocation, captive breeding programs, and so forth, have often been justified as being good for things like biodiversity or species preservation, ignoring the considerable harms that they have caused to wildlife, such as pain and suffering and disruption of social relationships. The traditional language of conservation biology fails to do justice to the ethical significance of animal cultures for the same reason that it has proven inadequate to the ethics of wildlife conservation generally: failing to take into account how and why cultures matter to animals themselves, and should as a result of that matter to us. Though not denying the value of such things as biodiversity (either as an intrinsic value in its own right or instrumentally for human or nonhuman interests) alternative approaches call for the recognition of individual animal welfare and agency (e.g., Edelblutte et al., 2023) and the self-determination of wildlife communities (e.g., Rizzolo & Bradshaw, 2021) as key values that conservation policy should take into account, and should often trump considerations like biodiversity. Similarly, recent work in animal ethics and political philosophy calls for viewing human relationships with wild animals in terms of relations between “self-determining political communities or “nations,” with rights of self-government, territorial sovereignty, or grounded jurisdiction” (Donaldson & Kymlicka, 2023, p. 630; see also, Papadopoulos, 2023).

What follows from this perspective? Compassionate conservation research and policy centered on animal wellbeing, agency, and self-determination can clearly benefit in a multitude of ways from greater understanding of the nature and role of social learning and socially learned behavior in animal populations, such as mitigating HWC and designing wildlife corridors or protected areas (Brakes et al., 2021, 2025). Cultural repertoire can also provide an important window into the vulnerabilities of particular populations *–* with cultural loss potentially serving as an early warning sign of population collapse (Kühl et al., 2019a; Malherbe et al., 2025) *–* as well as helping us to identify distinct populations that are most at risk (Whitehead et al., 2023). It is, however, misguided to single out particular populations for special protection or concerted effort *because* of their unique cultural behavioral repertoire. Limitations on resources and the scope of practically effective interventions make it inevitable that hard choices have to be made about where efforts should be best directed, but those choices should also be made in ethically defensible ways. Imagine that populations of humans were in a similarly precarious state, and we used features of their behavioral repertoire *–* unique tool-use or communicative practices, for instance *–* to prioritize them for assistance. Moreover, though cultural diversity and richness may indeed be an indicator of the health and self-determination of a population (Kühl et al., 2019a), we certainly should not think that the goal is the preservation of the behavioral repertoire itself, as if that were something independent of the animals that manifest it. The ideal goal should be maintaining the health and self-determination of as many individuals and populations as possible, irrespective of their cultural “complexity.” In this respect it is better to place the emphasis, as some researchers have done (see Brakes et al., 2025), on preserving cultural *capacity*, rather than on cultural practices or cultural diversity itself.

This perspective also casts some light on the ethics of intervening in the cultural lives of wildlife in the name of conservation. For example, researchers have begun to explore the possibility of restoring lost cultural practices, such as avian vocal cultures, via reintroductions of zoo-bred animals as cultural models or playback of recorded songs (Crates et al., 2025). Though there are many potential kinds of harm that need to be balanced here (such as the risk of population extirpation as a result of the loss of adaptive behavior), such interventions, along with some of those proposed for changing cultural behaviors implicated in HWC, do raise questions about the extent to which we may be inflicting harms on the agency and self-determination of animals by manipulating their cultures to serve our own goals (see also van Dooren et al., 2023). Such harms may, justifiably, have to be traded off against greater harms, but should nonetheless feature in our deliberations.

## Conclusion

Recall the public outreach argument: “By highlighting the cultural aspects of species, conservationists can create stronger emotional connections between the public and wildlife, which can lead to increased funding and political support for conservation initiatives” (Sueur, 2022, p. 99). It is worth thinking about why this might be so. What research on animal culture does is provide a window into the unique ways of life of animal communities *–* in particular, that populations of the same species have unique cultural identities, albeit not perhaps in such an elaborated form as human cultural communities, but unique, nonetheless. That creates emotional connections precisely because we are invited into these ways of living, and we may come to feel their loss (van Dooren et al., 2025). It seems strikingly inapposite to then frame the actual or potential loss of these ways of living in terms of losses to population resilience, biodiversity, adaptive potential, ecosystem services, or even “heritage.” Such language may serve an important pragmatic function when engaging with established conservation policy audiences and frameworks, and perhaps creates less chance of erroneously anthropomorphizing animals and their cultures, but it is nonetheless important not to gloss over the deeper ethical concern. This is certainly not to say that values such as biodiversity are not values worth caring about, but as Whitehead et al. (2004, p. 427) note, we do not appeal to such values to defend the ability of human beings to maintain their cultural traditions *–* presumably because the relevant humans would find that objectionable.

Existing arguments for conserving animal culture in the scientific literature clearly reveal a deep divide in our thinking about the value of humans and the value of animals. But the discovery of animal culture should be helping to close that gap. Cultural animals have interests in maintaining, innovating, learning, and demonstrating cultural behaviors because those are essential to their wellbeing and self-determination, independent of whatever adaptive utility the behaviors may have. These interests are among the morally relevant values that humans need to consider in acting. This leaves us with an ethical reason for caring about the persistence of animal cultures that goes beyond the traditional language of conservation biology, and understanding it helps us to think through some of the conceptual and ethical issues raised by the literature on animal culture conservation. We are, of course, under no illusions that such an ethical perspective is likely to be any more impactful than existing anthropocentric arguments for wildlife conservation when it comes to actually drawing resources and political will to make a real difference for endangered animal populations, and conservationists will need whatever arguments they can muster (anthropocentric and otherwise) in service of that end. Nonetheless, to show respect for their interests, those advocating for animals should also articulate the ethical arguments for protecting animals’ ability to continue their practices of culture building.

## Data Availability

Not applicable.

## Footnotes

1 As described on the UNESCO Natural World Heritage website, https://whc.unesco.org/en/natural-world-heritage/.

2 https://www.who.int/health-topics/one-health.

3 Another anthropocentric reason, though not one directly related to traditional conservation goals, is that preserving and documenting the cultural practices of animals, such as tool-related material culture in primates, is important for our scientific understanding of human evolution, by providing a testing ground for hypotheses about the nature and evolutionary origins of our ancestors’ cultural and psychological capacities (Kalan & Luncz, 2025).

4 As previously noted, the UNESCO World Heritage List also includes many “natural heritage” sites (such as the Great Barrier Reef and the Serengeti National Park), recognized as being of “outstanding universal value for humanity,” in virtue of their natural beauty and/or their exceptional biodiversity. See: https://whc.unesco.org/en/natural-world-heritage/
